# Abscess of Gutteral Pouch

**Published:** 1891-11

**Authors:** W. H. Ridge


### ABSCESS OF GUTTERAL POUCH.*

By W. H. Ridge, V.M.D.

Bay mare, six years old, well bred, weight about nine hundred and twenty-five pounds, served by Epaulet, September 23d, 1890. Taken sick August 3d, with symptoms of pharyngitis; another mare taken with similar symptoms recovered in two days The symptoms were severe chills, weather was warm, it took two heavy blankets on her before she stopped shaking ; I found after the chill the head lowered, nose out straight, champing the jaws continually (which lasted at intervals throughout her sickness), seemed anxious to eat, would chew the food, then quid it ; fluids, when swallowed, would be returned by nostrils, a gulping sound was produced when swallowing, then would cough ; the cough was moist and with rapel, she had a slight foamy discharge from nostril, the temperature was 103° F., which gradually lowered to normal on the 10th, to rise to 103° on the 22d, after a severe hemorrhage. Respirations were normal until the 25th, when they began to increase in frequency ; on 16th she had a free discharge of a stringy fœtid mucus, the breath was very fœtid after this until death. There were no swellings over the gutteral pouches or any part. No dyspnœa at any time. Vesicular murmur over both lungs. No points of dullness on percussion ; on turning did not show any stiffness except head and neck ; pulse was normal,

* Read before the Pennsylvania Veterinary Medical Association.

full, and strong, and not above forty-five until after the hemorrhage when it rose to ninety. Mucous membranes were rosy hue until after the hemorrhage, when they became pale. Bowels were normal ; urine appeared normal.

My diagnosis was at first pharyngitis ; later, post pharyngeal abscess. The mare was at first treated by heart sedatives, with Potass. Nit. in drinking water, followed in a few days by Hyd. Bell. Iod. blister to throat, and internal treatment of tonics, Dig., Acid Sulph., Potass. Iod. Dr. Rayner was called on the 14th. He found great tenderness over the third cervical vertebra, which he thought was due to an injury. Then we blistered the neck, and used hot packs. On the 16th she discharged about one pint of a stringy, fœtid substance from the nostrils, as if it came from some cavity ; after this we had a frothy discharge as before, except that it now remained fœtid. We now added Ferri. Sulph. to our treatment. On the 17th Dr. Rayner again saw her, when he confirmed my diagnosis of post-pharyngeal abscess, with a good prognosis. Temperature, now, 100.1° ; pulse, forty-two ; respiration, fourteen ; and eating three pints of oats besides oat meal gruel, and also grass and apples. But yet the head was held stiff as in poll evil, and if we attempted to move the head it gave her great pain. This condition lasted without change until the 21st when, in the night, she commenced with a violent hemorrhage, bleeding fully six quarts. When I arrived it had nearly stopped, but was flowing from nostrils and mouth, the mouth champing. The discharge was without cough, and did not discommode her as she would offer to eat while bleeding ; the blood was pure blood and without foam. Gave her Ext. Ergot when the hemorrhage stopped ; in about one hour she commenced coughing and sneezing, when the hemorrhage again commenced. We administered Morphia and Canabis Indica, followed by Ext. Ergot, fl. ʒiii every twenty minutes until she took ℥iii; when she had no more hemorrhage. Next morning gave her whiskey, milk and eggs, also Tinc. Bell., Cinchona Sulp., and Spts. Nits., emulsion. The next morning, the 22nd, Dr. Rayner again saw her when he approved of my treatment. The temperature now rose to 103° ; respiration remained fifteen ; pulse ninety, and weak but regular.

The prognosis was now grave, as the mare refused to take any nourishment. On the 23d she began to take gruel but with much more difficulty in swallowing it. Temperature rose to 104° ; respiration normal ; pulse eighty-four, weak. On the 24th Dr. Rayner again saw the mare; he thought it best to swab her throat out with a solution of Ferri. Sulph., which was done. Temperature 103°; pulse eighty; respiration fourteen. On the 25th, temperature 102.8 in vagina; respiration eighteen; pulse ninety; not eating, eye looked dull, countenance haggard, nose straight out, head in corner, legs cold, rectum patulous. Examined lungs, did not feel sure as to their condition. I also thought there was trouble in gutteral pouch and wanted to perform hyo-vertebrotomy; but, wishing advice, sent for Dr. Zuill, who found a small patch of pneumonia in right lung, and as he did not think there was anything wrong with the gutteral pouches, did not approve of operation. Applied blister to right side of chest; the left lung seemed all right. The prognosis was now more grave, as the mare had not enough strength to stand much of a pneumonia. On the 26th the temperature was 102.2 in vagina; respiration twenty-nine; pulse ninety-five, weak but regular; eyes sunken, mucous membrane dry, legs cold, crepitant, rales in middle and upper part of right lung; while we had mucous and blowing rales in the bottom, we now had crepitant rales in the left lung at the bottom. She stands in one place with the head in corner; breath same fœtor, but more so when disturbed.

On the evening of 28th, the mare died. Autopsy 10:30 A. M. next day. Temperature of weather, 80° in shade. Animal lying on left side; emaciated, but not tympanitic. The animal evidently had not struggled lying on peat moss. No discharge of fluids from any opening. Made incisions along the linea alba from pubis to submaxillary space, skinned down the neck, then sawed off inferior maxillary bone anterior to molars, disarticulated, and dissected down to gutteral pouch. Parotid and submaxillary salivary glands normal; opened right gutteral pouch, which was perfectly healthy; then opened larynx, which had a few minute points of inflammation. On opening the pharynx found the mucous membrane inflamed and points of ulceration; the teeth were stained black, and tongue normal. On opening the left gutteral pouch we found it about half full of cheesy fœtid pus and decomposed blood clot, and extensive ulcerated condition of the mucous membrane. The hyoid bone was ulcerated until it was disarticulated at its upper extremity; the ulceration extended through the occipito-atloid articulation. The cartilage of the occipital and atlas highly inflamed and ulcerated. The ulcerations had extended into the spinal canal, the fluid pushing the meninges from the bone. The sinuses were healthy. The third cervical was perfectly healthy, as well as the muscles over-lying it. Skined down the side, taking off the front leg with the skin, cutting the ribs top and bottom, leaving lungs exposed; cavity contained but very little fluid which was not fœtid. The pleura inflamed over the spots of pneumonia, bottom part of right lung; also anterior lobes becoming gangrenous, with pneumonia extending to middle. On section showed points of breaking down; left lung, hypostatic congestion with a commencing pneumonia in the lower part, with anterior lobes breaking down. Pericardium slightly inflamed; endocardium normal; chicken-fat clot in heart and aorta. Abdomen normal but pale; anæmic.

It is a question as to what caused the pneumonia and how long the anterior lobes could have been affected. Also, what was the primary trouble, whether due to injury breaking the hyoid, or pharyngitis ulcerating up the eustachian tube. Whether the pneumonia could be caused by Ergot constricting the vessels to such an extent as to produce gangrene; or, whether due to entrance of blood, producing traumatic pneumonia.

